# Gastrointestinal Stromal Tumors: A Retrospective Study at a Tertiary Care Center in Saudi Arabia in the Last Decade

**DOI:** 10.7759/cureus.64560

**Published:** 2024-07-15

**Authors:** Fahd Refai

**Affiliations:** 1 Department of Pathology, King Abdulaziz University Hospital, King Abdulaziz University, Jeddah, SAU

**Keywords:** tumor characteristics, risk assessment, epidemiology, saudi arabia, gastrointestinal stromal tumors

## Abstract

Introduction: Gastrointestinal stromal tumors (GISTs) are a significant subset of mesenchymal tumors primarily found in the gastrointestinal tract, impacting diagnostic and therapeutic approaches. Understanding their epidemiology is crucial for improving patient care and advancing treatment strategies.

Methodology: Our study at a Saudi tertiary hospital analyzed 50 patients with GIST, focusing on demographics, tumor locations, and risk assessments. We examined predictors of tumor size, including mitosis frequency, and assessed the impact of anatomical location and risk on clinical outcomes using RStudio software (Posit, Boston, MA).

Results: Among 50 patients with GIST, 36 (72.0%) were male with a median age of 60.5 years, and most tumors (33, 66.0%) were in the stomach. Risk assessments categorized tumors as follows: 20 (40.0%) low risk, 12 (24.0%) high risk, 7 (14.0%) moderate risk, 7 (14.0%) very low risk, and 4 (8.0%) no risk. Most tumors were low-grade (41, 82.0%) and nonmetastatic (47, 94.0%), predominantly spindle cell type (37, 74.0%). Tumor size varied significantly across risk categories: high-risk tumors averaged 10.3 cm versus 0.5 cm for no risk and 3.5 cm for very low risk (*P* < 0.001). Mitosis frequency differed significantly by risk category and tumor grade (*P* < 0.001). Tumor grade varied notably with risk categories and morphologic types, especially high-grade tumors in high-risk groups (8, 66.7%) and epithelioid tumors (2, 100%). Multivariable analysis identified predictors of tumor size: anatomical location (extra-GI, intra-abdominal; beta = 7.08, *P* = 0.011) and risk assessment (low risk, beta = 6.91, *P* = 0.001; moderate risk, beta = 11.2, *P* < 0.001; high risk, beta = 8.93, *P* < 0.001). Liver metastasis did not differ significantly across gender, anatomical location, risk assessment, or tumor grade.

Conclusions: In Saudi Arabia, GISTs predominantly affect males and are primarily located in the stomach. Our findings highlight significant variations in tumor size and grade based on risk assessments and anatomical location. Most GISTs were low-grade, nonmetastatic, and spindle cell type, emphasizing the need for enhanced research to improve diagnostics, tailor treatments, and optimize outcomes in the region.

## Introduction

Gastrointestinal stromal tumors (GISTs) are the predominant type of mesenchymal tumors in the GI tract. They constitute 80% of all such tumors and represent a small fraction, between 0.1% and 3%, of all GI cancers [1,2]. Around 30% of GISTs are cancerous [3]. GISTs can form throughout the entire gastrointestinal tract, but they are primarily found in the stomach (60%) and the small intestine (20%-30%) [4-6]. GISTs can occasionally develop outside the GI tract, often in areas like the omentum, mesentery, or retroperitoneum. Initially categorized as smooth muscle tumors in the 1980s, advances in immunohistochemistry and the identification of gain-of-function mutations over the past two decades have established GISTs as distinct entities [7].

GISTs mostly have well-defined boundaries and commonly develop within the muscularis propria layer of the gastrointestinal tract. Their size can vary, with high-risk GISTs having a median tumor size of about 8.9 cm. [8,9]. The majority of GISTs typically display intense and widespread staining in the cytoplasm for KIT, while a smaller number may show a staining pattern characterized by dots or a membranous distribution [10-12]. The intensity and manner in which KIT is detected immunohistochemically do not influence the probability of responding to treatment [13].

Understanding the epidemiology of GIST in Saudi Arabia is crucial, especially given the minimal data available in the literature. It helps recognize patterns and prevalence, improving diagnostic practices and raising awareness among healthcare providers and patients. Specific epidemiological data can reveal variations in GIST subtypes and treatment responses, aiding in developing tailored treatment strategies and effective healthcare resource allocation. Additionally, it supports the formulation of public health policies, enhances patient outcomes, and guides research efforts for new diagnostic tools and therapies. At a tertiary hospital in Saudi Arabia, our study aimed to comprehensively analyze demographic characteristics, tumor locations, risk assessments, and tumor grades among 50 patients diagnosed with GIST. We focused on understanding correlations between numerical variables and differences across categorical groups, particularly examining tumor size and mitosis frequency across varying risk assessment categories. Additionally, our research delved into identifying predictors of tumor size through rigorous univariable and multivariable regression analyses, highlighting the significance of anatomical location and risk assessment in shaping clinical outcomes for these patients.

## Materials and methods

The study enrolled patients diagnosed with GIST at King Abdulaziz University Hospital between 2013 and 2023. Inclusion criteria were confirmed GIST cases through immunohistochemical staining and microscopic examination with complete clinical and demographic data. Exclusion criteria were applied to patients lacking comprehensive clinical or demographic information. Ethical approval was obtained from the Research Ethics Committee of King Abdulaziz University, Faculty of Medicine (Reference No. 207-27, Unit of Biomedical Ethics). The data were extracted from medical records while maintaining patient confidentiality. We diagnose GIST patients and classify them for risk assessment based on the GEIS Guidelines [14]. For extra-gastrointestinal GIST, classification was conducted according to the National Institutes of Health (NIH) criteria, as modified by the Joensuu Risk Stratification [15].

Statistical analysis

The statistical analysis was carried out using RStudio software (R version 4.3.1; Posit, Boston, MA). Categorical variables were described using frequencies and percentages. The Shapiro-Wilk test was used to assess the normality of numerical variables. Results showed significant *P*-values (*P* = 0.022, *P* = 0.005, and *P* < 0.001 for age, tumor size, and mitosis frequency, respectively). This indicated non-normally distributed variables. Numerical variables were summarized as median (interquartile range [IQR]). Statistical differences in patients’ groups based on categorical variables were assessed using a Fisher’s exact test. Spearman’s correlation test was applied to evaluate the relationships between tumor size, age, and mitosis frequency. Differences in numerical variables across categorical groups were assessed using the Wilcoxon rank sum test or the Kruskal-Wallis rank sum test. Univariable and multivariable linear regression analyses were conducted to identify significant predictors of tumor size. The default significance level was set at *P* < 0.05.

## Results

Description of the characteristics and outcomes of patients

**Figure 1:**
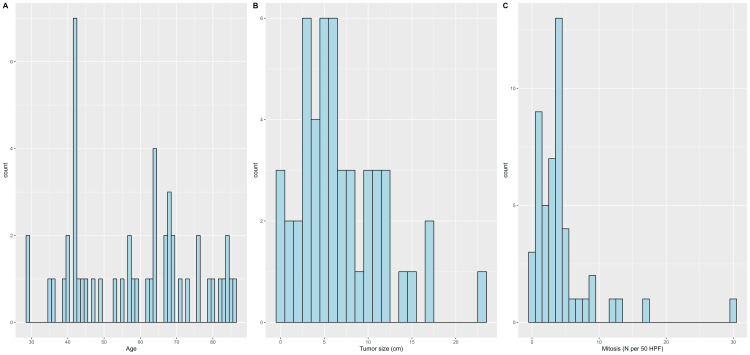
Frequency distributions of patients’ (A) age, (B) tumor size, and (C) mitosis.

The study included 50 patients, with 36 (72.0%) males and 14 (28.0%) females. The anatomical location of the tumors was primarily in the stomach (33, 66.0%), followed by the jejunum (7, 14.0%). Regarding risk assessment, 20 (40.0%) tumors were classified as low risk, 12 (24.0%) as high risk, 7 (14.0%) as moderate risk, 7 (14.0%) as very low risk, and 4 (8.0%) had no risk. Most tumors were low grade (41, 82.0%), with 47 (94.0%) having no metastasis. The majority of tumors were of the spindle cell type (37, 74.0%), followed by mixed (11, 22.0%) and epithelioid (2, 4.0%) types (Table 1). The median age of the patients was 60.5 years (IQR, 42.0-69.0). The median tumor size was 6.0 cm (IQR, 3.5-10.0), and the median mitosis count per high-power field was 4.0 (IQR, 2.0-4.8; Table 2). The frequency distributions of numerical variables are depicted in Figure 1.

**Table 1:** Description of the categorical variables.

Characteristic	Description, *n* (%)
Gender	
Male	36 (72.0%)
Female	14 (28.0%)
Anatomical location	
Stomach	33 (66.0%)
Duodenum	4 (8.0%)
Jejunum	7 (14.0%)
Rectum	2 (4.0%)
Extra-gastrointestinal organs (intra-abdominal)	2 (4.0%)
Extra-gastrointestinal organs (pelvis)	2 (4.0%)
Risk assessment	
None	4 (8.0%)
Very low	7 (14.0%)
Low	20 (40.0%)
Moderate	7 (14.0%)
High	12 (24.0%)
Grade	
Low	41 (82.0%)
High	9 (18.0%)
Presence of metastasis	
None	47 (94.0%)
Liver metastasis	3 (6.0%)
Morphologic type	
Spindle	37 (74.0%)
Epithelioid	2 (4.0%)
Mixed	11 (22.0%)

**Table 2:** Description of the numerical variables. IQR, interquartile range; SD, standard deviation

Characteristic	Median (IQR)	Mean ± SD	Min-Max
Age	60.5 (42.0-69.0)	58.5 ± 16.4	29.0-86.0
Tumor size (cm)	6.0 (3.5-10.0)	7.0 ± 4.9	0.4-23.0
Mitosis (N per 50 HPF)	4.0 (2.0-4.8)	4.5 ± 5.0	0.0-30.0

Differences in numerical variables across categorical groups

There were no significant differences in participants’ ages across different categories (Table 3). Regarding tumor size, results showed a significant difference across risk assessment categories, with medians of 0.5 cm (IQR, 0.5-0.5) for no risk, 3.5 cm (IQR, 2.5-4.5) for very low risk, 5.8 cm (IQR, 3.8-7.6) for low risk, 11.5 cm (IQR, 6.3-11.8) for moderate risk, and 10.3 cm (IQR, 7.7-12.1) for high risk (*P* < 0.001; Table 4). Similarly, mitosis frequency showed significant differences across risk assessment categories (*P* < 0.001) and tumor grade (*P* < 0.001; Table 5).

**Table 3:** The differences in age across different categorical groups. *P*-values are based on a Wilcoxon rank-sum test or a Kruskal-Wallis rank-sum test.

Characteristic	Age	p-value
Gender		0.325
Male	64.0 (43.5-69.5)	
Female	55.0 (42.0-66.8)	
Anatomical location		0.097
Stomach	64.0 (49.0-71.0)	
Duodenum	63.0 (43.0-82.5)	
Jejunum	42.0 (32.0-53.0)	
Rectum	49.5 (42.8-56.3)	
Extra-gastrointestinal organs (intra-abdominal)	52.0 (46.0-58.0)	
Extra-gastrointestinal organs (pelvis)	64.0 (53.5-74.5)	
Risk assessment		0.865
None	63.0 (54.0-68.3)	
Very low	68.0 (55.0-71.0)	
Low	60.5 (43.5-70.0)	
Moderate	42.0 (42.0-81.0)	
High	59.0 (41.3-64.0)	
Grade		0.676
Low	58.0 (42.0-69.0)	
High	64.0 (55.0-64.0)	
Presence of metastasis		0.870
None	62.0 (42.0-69.0)	
Liver metastasis	45.0 (44.5-58.0)	
Morphologic type		0.374
Spindle	57.0 (42.0-69.0)	
Epithelioid	59.5 (57.3-61.8)	
Mixed	67.0 (55.5-73.5)	

**Table 4:** The differences in tumor size across different categorical groups. *P*-values are based on a Wilcoxon rank-sum test or a Kruskal-Wallis rank-sum test.

Characteristic	Tumor size (cm)	*P*-value
Gender		0.871
Male	5.5 (3.6-10.4)	
Female	6.7 (3.6-9.5)	
Anatomical location		0.101
Stomach	6.0 (3.5-9.4)	
Duodenum	4.0 (3.0-5.3)	
Jejunum	4.5 (3.5-8.8)	
Rectum	11.3 (10.9-11.6)	
Extra-gastrointestinal organs (intra-abdominal)	15.5 (14.8-16.3)	
Extra-gastrointestinal organs (pelvis)	7.5 (7.2-7.9)	
Risk assessment		<0.001
None	0.5 (0.5-0.5)	
Very low	3.5 (2.5-4.5)	
Low	5.8 (3.8-7.6)	
Moderate	11.5 (6.3-11.8)	
High	10.3 (7.7-12.1)	
Grade		0.088
Low	5.5 (3.4-8.2)	
High	10.0 (6.0-12.0)	
Presence of metastasis		0.487
None	6.0 (3.6-10.3)	
Liver metastasis	5.0 (3.3-6.5)	
Morphologic type		0.174
Spindle	5.5 (3.0-9.4)	
Epithelioid	12.5 (11.3-13.8)	
Mixed	6.0 (4.9-9.8)	

**Table 5:** The differences in mitosis frequency across different categorical groups. *P*-values are based on a Wilcoxon rank-sum test or a Kruskal-Wallis rank-sum test.

Characteristic	Mitosis frequency	*P*-value
Gender		0.200
Male	3.0 (1.0-4.3)	
Female	4.0 (3.0-4.8)	
Anatomical location		0.207
Stomach	3.0 (1.0-5.0)	
Duodenum	3.0 (2.8-3.3)	
Jejunum	4.0 (2.0-4.0)	
Rectum	14.5 (13.3-15.8)	
Extra-gastrointestinal organs (intra-abdominal)	6.0 (4.5-7.5)	
Extra-gastrointestinal organs (pelvis)	4.5 (4.3-4.8)	
Risk assessment		<0.001
None	1.0 (0.8-1.3)	
Very low	3.0 (1.5-4.5)	
Low	3.0 (2.0-4.0)	
Moderate	1.0 (1.0-4.0)	
High	8.5 (4.8-12.3)	
Grade		<0.001
Low	3.0 (1.0-4.0)	
High	9.0 (8.0-13.0)	
Presence of metastasis		0.214
None	4.0 (1.5-4.0)	
Liver metastasis	8.0 (5.0-10.5)	
Morphologic type		0.162
Spindle	3.0 (2.0-5.0)	
Epithelioid	7.5 (6.8-8.3)	
Mixed	4.0 (1.0-4.0)	

Results of bivariate correlations between numerical variables

**Figure 2:**
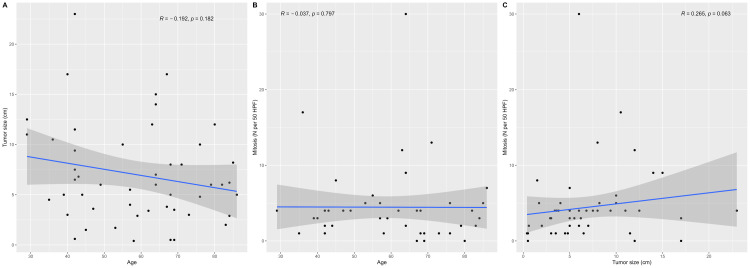
Scatterplots depicting the correlations between (A) tumor size and age, (B) mitosis frequency and age, and (C) mitosis frequency and tumor size. *P*-values are based on Spearman’s correlation tests because all the variables were non-normally distributed according to the Shapiro-Wilk test, including age (*P* = 0.022), mitosis frequency (*P* < 0.001), and tumor size (*P* = 0.005).

The scatterplot between tumor size and age (Figure 2A) shows a weak negative correlation, indicating no significant relationship between these variables. The scatterplot between mitosis frequency and age (Figure 2B) also shows a weak correlation, showing no significant relationship. In contrast, the scatterplot between mitosis frequency and tumor size (Figure 2C) indicated no significant correlation.

Statistical differences in patients' groups

Based on the risk assessment, there was a significant difference in tumor grade across different risk assessment categories (*P* < 0.001). High-grade tumors were more prevalent in the high-risk group (66.7%) compared to none in the no risk, very low risk, and low-risk groups. All tumors in the no-risk, very low risk, and low-risk groups were of low grade (Table 6).

**Table 6:** Statistical differences in patients’ groups based on risk assessment categories. Fisher's exact test.

Characteristic	None (N = 4)	Very low (*N* = 7)	Low (*N* = 20)	Moderate (*N* = 7)	High (*N* = 12)	*P*-value
Gender, *n* (%)						0.883
Male	4 (100.0%)	5 (71.4%)	14 (70.0%)	5 (71.4%)	8 (66.7%)	
Female	0 (0.0%)	2 (28.6%)	6 (30.0%)	2 (28.6%)	4 (33.3%)	
Anatomical location, *n* (%)						0.451
Stomach	4 (100.0%)	7 (100.0%)	12 (60.0%)	5 (71.4%)	5 (41.7%)	
Duodenum	0 (0.0%)	0 (0.0%)	3 (15.0%)	1 (14.3%)	0 (0.0%)	
Jejunum	0 (0.0%)	0 (0.0%)	4 (20.0%)	1 (14.3%)	2 (16.7%)	
Rectum	0 (0.0%)	0 (0.0%)	0 (0.0%)	0 (0.0%)	2 (16.7%)	
Extra-gastrointestinal organs (intra-abdominal)	0 (0.0%)	0 (0.0%)	1 (5.0%)	0 (0.0%)	1 (8.3%)	
Extra-gastrointestinal organs (pelvis)	0 (0.0%)	0 (0.0%)	0 (0.0%)	0 (0.0%)	2 (16.7%)	
Grade, *n* (%)						<0.001
Low	4 (100.0%)	7 (100.0%)	20 (100.0%)	6 (85.7%)	4 (33.3%)	
High	0 (0.0%)	0 (0.0%)	0 (0.0%)	1 (14.3%)	8 (66.7%)	
Presence of metastasis, *n* (%)						0.577
None	4 (100.0%)	7 (100.0%)	19 (95.0%)	7 (100.0%)	10 (83.3%)	
Liver metastasis	0 (0.0%)	0 (0.0%)	1 (5.0%)	0 (0.0%)	2 (16.7%)	
Morphologic type, *n* (%)						0.297
Spindle	4 (100.0%)	6 (85.7%)	14 (70.0%)	4 (57.1%)	9 (75.0%)	
Epithelioid	0 (0.0%)	0 (0.0%)	0 (0.0%)	0 (0.0%)	2 (16.7%)	
Mixed	0 (0.0%)	1 (14.3%)	6 (30.0%)	3 (42.9%)	1 (8.3%)	

Based on the morphologic type, there was a significant difference in tumor grade across different morphologic types (*P* = 0.034). High-grade tumors were more prevalent among epithelioid tumors (100%) compared to spindle (16.2%) and mixed (9.1%) tumors (Table 7). Regarding metastasis, there were no significant differences observed in gender (*P* = 0.550), anatomical location (*P* = 0.533), risk assessment (*P* = 0.577), tumor grade (*P* = 0.080), or morphologic type (*P* = 0.604) between patients with and without liver metastasis (Table 8). 

**Table 7:** Statistical differences in patients' groups based on the morphologic type. Fisher's exact test.

Characteristic	Spindle (*N *= 37)	Epithelioid (*N *= 2)	Mixed (*N *= 11)	*P*-value
Gender, *n* (%)				0.203
Male	24 (64.9%)	2 (100.0%)	10 (90.9%)	
Female	13 (35.1%)	0 (0.0%)	1 (9.1%)	
Anatomical location, *n* (%)				0.987
Stomach	22 (59.5%)	2 (100.0%)	9 (81.8%)	
Duodenum	3 (8.1%)	0 (0.0%)	1 (9.1%)	
Jejunum	6 (16.2%)	0 (0.0%)	1 (9.1%)	
Rectum	2 (5.4%)	0 (0.0%)	0 (0.0%)	
Extra-gastrointestinal organs (Intra-abdominal)	2 (5.4%)	0 (0.0%)	0 (0.0%)	
Extra-gastrointestinal organs (Pelvis)	2 (5.4%)	0 (0.0%)	0 (0.0%)	
Risk assessment, *n* (%)				0.297
None	4 (10.8%)	0 (0.0%)	0 (0.0%)	
Very low	6 (16.2%)	0 (0.0%)	1 (9.1%)	
Low	14 (37.8%)	0 (0.0%)	6 (54.5%)	
Moderate	4 (10.8%)	0 (0.0%)	3 (27.3%)	
High	9 (24.3%)	2 (100.0%)	1 (9.1%)	
Grade, *n* (%)				0.034
Low	31 (83.8%)	0 (0.0%)	10 (90.9%)	
High	6 (16.2%)	2 (100.0%)	1 (9.1%)	
Presence of metastasis, *n* (%)				0.604
None	35 (94.6%)	2 (100.0%)	10 (90.9%)	
Liver metastasis	2 (5.4%)	0 (0.0%)	1 (9.1%)	

**Table 8:** Statistical differences in patients' groups based on metastasis. We could not fit a multivariable model due the existence of zero frequencies in multiple categories. Fisher's exact test.

Characteristic	None (*N* = 47)	Liver metastasis (*N* = 3)	*P*-value
Gender, *n* (%)			0.550
Male	33 (70.2%)	3 (100.0%)	
Female	14 (29.8%)	0 (0.0%)	
Anatomical location, *n* (%)			0.533
Stomach	31 (66.0%)	2 (66.7%)	
Duodenum	3 (6.4%)	1 (33.3%)	
Jejunum	7 (14.9%)	0 (0.0%)	
Rectum	2 (4.3%)	0 (0.0%)	
Extra-gastrointestinal organs (intra-abdominal)	2 (4.3%)	0 (0.0%)	
Extra-gastrointestinal organs (pelvis)	2 (4.3%)	0 (0.0%)	
Risk assessment, *n* (%)			0.577
None	4 (8.5%)	0 (0.0%)	
Very low	7 (14.9%)	0 (0.0%)	
Low	19 (40.4%)	1 (33.3%)	
Moderate	7 (14.9%)	0 (0.0%)	
High	10 (21.3%)	2 (66.7%)	
Grade, *n* (%)			0.080
Low	40 (85.1%)	1 (33.3%)	
High	7 (14.9%)	2 (66.7%)	
Morphologic type, *n* (%)			0.604
Spindle	35 (74.5%)	2 (66.7%)	
Epithelioid	2 (4.3%)	0 (0.0%)	
Mixed	10 (21.3%)	1 (33.3%)	

Univariable and multivariable regression analyses of tumor size

In the univariable analysis, significant associations with tumor size were found for anatomical location (extra-gastrointestinal organs, intra-abdominal; beta = 8.8; 95% confidence interval [CI], 2.2-15; *P* = 0.012) and risk assessment (low risk, beta = 6.2; 95% CI, 1.9-10; *P* = 0.007; moderate risk, beta = 10; 95% CI, 5.4-15; *P* < 0.001; high risk, beta = 9.1; 95% CI, 4.6-14; *P* < 0.001). In the multivariable analysis, significant predictors of tumor size were anatomical location (extra-gastrointestinal organs, intra-abdominal; beta = 7.08, 95% CI, 1.84-12.3; *P* = 0.011) and risk assessment (low risk; beta = 6.91; 95% CI, 2.97-10.9; *P* = 0.001; moderate risk, beta = 11.2, 95% CI, 6.78-15.7; *P* < 0.001; high risk, beta = 8.93; 95% CI, 4.53-13.3, *P* < 0.001; Table 9).

**Table 9:** Univariable and multivariable regression analyses of tumor size. CI, confidence interval

Characteristic	Univariable regression			Multivariable regression		
	Beta	95% CI	*P*-value	Beta	95% CI	*P*-value
Gender						
Male	Reference	Reference				
Female	-0.48	-3.5 to 2.5	0.759			
Age	-0.06	-0.14 to 0.02	0.150			
Anatomical location						
Stomach	Reference	Reference		Reference	Reference	
Duodenum	-2.5	-7.3 to 2.3	0.317	-4.26	-8.11 to -0.42	0.036
Jejunum	-0.35	-4.1 to 3.4	0.856	-2.26	-5.30 to 0.78	0.152
Rectum	4.6	-2.0 to 11	0.182	1.82	-3.80 to 7.44	0.529
Extra-gastrointestinal organs (intra-abdominal)	8.8	2.2 to 15	0.012	7.08	1.84 to 12.3	0.011
Extra-gastrointestinal organs (pelvis)	0.81	-5.8 to 7.4	0.811	-1.93	-7.55 to 3.69	0.504
Mitosis (N per 50 HPF)	0.14	-0.13 to 0.41	0.324			
Risk assessment						
None	Reference	Reference		Reference	Reference	
Very low	3.0	-1.9 to 7.8	0.242	2.96	-1.41 to 7.33	0.192
Low	6.2	1.9-10	0.007	6.91	2.97-10.9	0.001
Moderate	10	5.4-15	<0.001	11.2	6.78-15.7	<0.001
High	9.1	4.6-14	<0.001	8.93	4.53-13.3	<0.001
Grade						
Low	Reference	Reference				
High	2.6	-0.90 to 6.0	0.154			
Presence of metastasis						
None	Reference	Reference				
Liver metastasis	-2.3	-8.0 to 3.4	0.428			
Morphologic type						
Spindle	Reference	Reference				
Epithelioid	5.9	-0.88 to 13	0.094			
Mixed	1.0	-2.2 to 4.2	0.543			

## Discussion

The Dutch GIST Registry cohort consisted of 1,425 patients, with an almost equal sex distribution: 46% female and 54% male patients [16]. In contrast, our study included 50 patients, with a significantly higher proportion of males: 36 (72%) compared to 14 (28%) females. According to Rong et al., among patients with GIST, there was a slight predilection for men to develop the disease compared to women, with a distribution of 54% and 46%, respectively [17].

In our cohort of patients with GIST, the majority of tumors (33, 66%) were located in the stomach. This distribution aligned well with existing literature, which also identifies the stomach as the most common site for GISTs [18-20].

The median age of 60.5 years for our patients diagnosed with GISTs in our study places them within the middle-age bracket, generally considered to range from 45 to 65 years. This age statistic serves as a central indicator of the age distribution among our study population, underscoring the typical age group most frequently affected by GISTs. Supporting this observation, previous research by Farag et al. and Joensuu et al. has found that the highest incidence of GISTs occurs in individuals aged 60 to 74 years, with a significant number of cases also observed in those aged 75 and older. This finding was consistent with the age profile seen in our data [21].

Based on these clinicopathological parameters, the NIH and the National Comprehensive Cancer Network (NCCN) have developed systems to predict GIST behavior through a risk assessment framework that categorizes tumors as very low risk, low risk, intermediate risk, or high risk [22]. In our analysis of GIST risk assessment, we found that 20 (40.0%) tumors were categorized as low risk, indicating a relatively favorable prognosis. Conversely, 12 (24.0%) were classified as high risk, suggesting a more aggressive disease course requiring intensive management. Moderate-risk tumors accounted for 7 (14.0%) cases, while very low-risk tumors and those with no identified risk constituted 7 (14.0%) and 4 (8.0%), respectively. In contrast, previous studies, such as those by Brabec et al. and Brady-West and Blake, have predominantly reported higher proportions of tumors falling into the high-risk category [23,24].

In our cohort, a significant proportion of GIST tumors were identified as low grade, accounting for the majority at 41 (82.0%). This finding underscores the predominantly indolent nature of these tumors within our study population. Moreover, a striking 47 (94.0%) of these cases exhibited no evidence of metastasis. These statistics highlight the generally favorable prognosis associated with low-grade GIST tumors, affirming their typically slow progression and less aggressive clinical course. Such observations underscore the importance of early detection and tailored management strategies aimed at optimizing patient outcomes in GIST management.

In our study, spindle cell tumors were the most predominant histological subtype, followed by mixed-type tumors and epithelioid tumors, respectively. This pattern contrasts with the commonly accepted classifications in the literature, where GISTs are typically categorized into three main histological subtypes: spindle cell type (most common, approximately 70%), epithelioid type (20%-25%), and mixed spindle cell and epithelioid types. This deviation highlights the unique distribution observed in our cohort, with mixed-type tumors occupying the second position in prevalence [25-27]. Our findings underscore the heterogeneity in GIST presentation and highlight the importance of risk assessment and histological subtypes in guiding personalized treatment approaches and predicting patient outcomes.

In our study of patients with GIST, we identified significant variations in tumor size across different risk assessment categories based on the modified NIH consensus criteria [28]. Tumors classified as no risk tended to be smaller, while those categorized as very low, low, moderate, and high risk demonstrated progressively larger median sizes. This finding highlights the clear association between risk assessment according to these criteria and tumor size within our dataset.

Additionally, we found significant differences in mitosis frequency across risk assessment categories and tumor grades. These findings highlight the histopathological diversity among GISTs and emphasize the importance of evaluating both tumor size and mitotic activity in guiding clinical management and predicting outcomes for patients with GISTs.

Our analysis revealed that gender, tumor morphological type, and anatomical location (gastrointestinal or extra-gastrointestinal) showed no significant associations with age, tumor size, or mitosis frequency. This suggests that these factors may not independently influence these key characteristics in our study cohort of patients with GIST. These findings underscore the complexity of GIST pathology, where risk assessment and other clinical factors may play more crucial roles in determining disease progression and management strategies.

In our study of GISTs, bivariate correlation analyses revealed nuanced relationships among key numerical variables. The scatterplot examining tumor size against age showed a weak negative correlation, suggesting a tendency for slightly smaller tumors in older patients, although this relationship was not statistically significant. Similarly, the scatterplot of mitosis frequency against age displayed a weak correlation, indicating that age does not notably influence the rate of cell division within GISTs. Importantly, the scatterplot of mitosis frequency against tumor size revealed no significant correlation, suggesting that tumor size and mitotic activity vary independently within our study cohort. These findings underscore the complex interplay among age, tumor size, and mitotic activity in GIST pathology, emphasizing the need for comprehensive evaluation of multiple factors in understanding disease characteristics and guiding clinical management strategies.

In the context of evaluating tumor characteristics and their association with risk factors, it was observed that tumor grade distribution significantly varied among different risk assessment categories. High-grade tumors were predominantly found in patients classified under the high-risk category, while those categorized as no risk, very low risk, or low risk exclusively presented with low-grade tumors. This suggests a clear correlation between higher risk assessment and increased tumor grade. Additionally, when analyzing tumors based on morphologic types, epithelioid tumors were more frequently high-grade compared to spindle and mixed tumors, indicating a significant relationship between morphologic type and tumor grade. Despite these findings, no significant differences were detected in the occurrence of liver metastasis when considering factors such as gender, anatomical location, risk assessment category, tumor grade, or morphologic type, suggesting that these variables may not be major determinants of metastatic behavior in this context.

We found a significant correlation between tumor size and both anatomical location and risk assessment. Specifically, tumors situated in extra-gastrointestinal intra-abdominal sites tend to manifest larger sizes, while higher risk assessments align with increased tumor dimensions. This is consistent with findings from Ghartimagar et al., where they observed that clinical presentations of GISTs were heavily influenced by tumor location and size. Mesenteric extra-gastrointestinal stromal tumors (E-GISTs), for instance, may remain asymptomatic for an extended period as they have ample space to grow before clinical symptoms become apparent [29].

In the multivariable analysis, anatomical location and risk assessment remain significant predictors of tumor size. This means that their influence on tumor size is independent of the other factors being considered. For example, even when considering other variables, tumors in extra-gastrointestinal, intra-abdominal organs are typically larger, and tumors classified as moderate or high risk are larger as well.

Limitations of our study include the relatively small sample size of 50 patients, which may restrict the generalizability of findings to broader populations. Being conducted at a single center, the study's results may not fully capture the diverse clinical presentations and outcomes observed in different healthcare settings.

## Conclusions

In line with previous research, our study provides a thorough examination of GISTs in our cohort, highlighting the majority of patients' male gender and the location of the stomach tumor. The majority of cancers were found to be low-grade and nonmetastatic, indicating a generally good prognosis. Histological analysis identified spindle cell tumors as the most prevalent subtype, with mixed-type tumors also prominently observed, highlighting the diverse nature of GIST presentations.

Significant variations in tumor size and mitosis frequency across different risk categories underscore their critical role in risk assessment and prognosis. These results support the significance of these factors in identifying the degree of aggression and possible consequences of GISTs. However, gender, tumor morphology, and anatomical location did not exhibit notable associations with these key characteristics, illustrating the multifaceted nature of GIST pathology and indicating that these factors alone may not directly influence prognosis.

The significance of customized treatment plans founded on reliable risk assessment techniques is highlighted by our findings. Individualized treatment plans that consider tumor size and mitotic rate are crucial for optimizing patient outcomes. Further research is essential to refine clinical approaches, identify additional prognostic factors, and enhance outcomes for GIST patients in Saudi Arabia and beyond. Continued investigation will be vital in improving our understanding of GISTs and developing more effective, personalized therapies.
